# Hematopoietic Aging Biomarkers in *Peromyscus leucopus* Mice

**DOI:** 10.4172/2329-8847.1000169

**Published:** 2017-02-06

**Authors:** Zenghua Lin, Sachiko Kajigaya, Xingmin Feng, Jichun Chen, Neal S Young

**Affiliations:** 1 Hematology Branch, National Heart, Lung, and Blood Institute, National Institutes of Health, Bethesda, MD USA; 2 Hematology Department, Affiliated Hospital of Nantong University, Nantong, Jiangsu, China

## Abstract

We analyzed hematopoietic phenotypes in *Peromyscus leucopus* (PL) mice at young (2–9 months), middle (22–23 months) and old (33–46 months) ages aimed at characterizing age-associated changes in this unique rodent species. We found a significantly higher number of monocytes in old PL mice in peripheral blood, and higher proportions of CD44^+^ cells in blood, spleen and bone marrow in old PL mice than in middle and young counterparts. We conclude that elevated blood monocyte counts and up-regulated hematopoietic cell CD44 expression are two useful aging biomarkers for PL mice.

## Introduction and Methods

*Peromyscus leucopus* (PL) mice are the most populous rodent species that reside in northeastern United States [1]. These animals, nicknamed “white-footed” mice, have distinctive high agility and long lifespans and are taxonomically distant from the frequently-used laboratory mice *Mus. musculus* (MM) [2–5]. Relative to MM mice, PL mice have fewer platelets and more monocytes [6] and their peripheral blood mononuclear cells have drastically shorter telomeres [7]. The characteristic short telomere and long lifespan in PL mice provide an interesting combination that favors the utility of PL mice as a potential model for aging studies.

Age-related phenotypic changes have been well studied in MM mouse models. Aging was found to cause hematopoietic lineage bias toward myeloid cells [8]. One specific change with age is the expression of CD44, a transmembrane glycoprotein present on the surface of many cell types. Proportions of CD44^+^ cells are significantly higher in CD4 and CD8 T cells in old mice, which correspond to memory T cell accumulation [9–11] and gamma interferon (IFN-γ)-producing T cell enrichment [12]. Changes in CD44 expression have also been described in other species [13,14]. Age-associated increase in CD44^+^ CD4 cells are found in the spleen of Dark Agouti rats [15]. In rat aorta endothelium, CD44 expression increases in an age-dependent fashion [16].

In the current study, we obtained PL mice from Peromyscus Genetic Stock Center (PGSC) at the University of South Carolina (Columbia, SC) to study age-associated changes in hematopoietic tissues. The PL mouse stock was originated from 38 founders captured near Linville Fall, North Carolina following restricted random breeding in captivity avoiding sister-brother mating [17]. Young (2–9 months) and middle-age (22–23 months) PL mice were produced at National Institutes of Health from breeders obtained from PGSC, while old PL mice (33–46 months) were originally obtained from PGSC at young age. All animal studies were approved by the Animal Care and Use Committee at the National Heart, Lung, and Blood Institute.

Blood was collected from retro-orbital sinus and complete blood counts (CBCs) were analyzed using a HemaVet 950 analyzer (Drew Scientific, Inc. CT). Mice were euthanized by CO_2_ inhalation from a compressed source. Bone marrow (BM) cells were extracted from bilateral tibiae and femurs, while spleen single cell suspension was obtained by homogenizing the spleen with a Kimble tissue grinder. BM and spleen cells were filtered through 95 μM nylon mesh and counted using a Vi-Cell counter (Coulter Cooperation, FL). Red blood cells (RBCs) in blood, BM, and spleen cells were lysed with ACK lysing buffer. Nucleated cells were stained with allophycocyanin (APC)-conjugated anti-mouse CD44 (clone 1M7) antibody (BD Bioscience, San Diego, CA) on ice for 30 minutes. Stained cells were acquired and analyzed on FACSCanto II flow cytometer using the FACSDiva software (Becton Dickson, San Diego CA). Data from CBC and flow cytometry were analyzed by GraphPad Prism 6 statistical software through variance analyses and multiple comparisons, and were shown as means with standard errors.

## Results and Discussion

**Figure 1:**
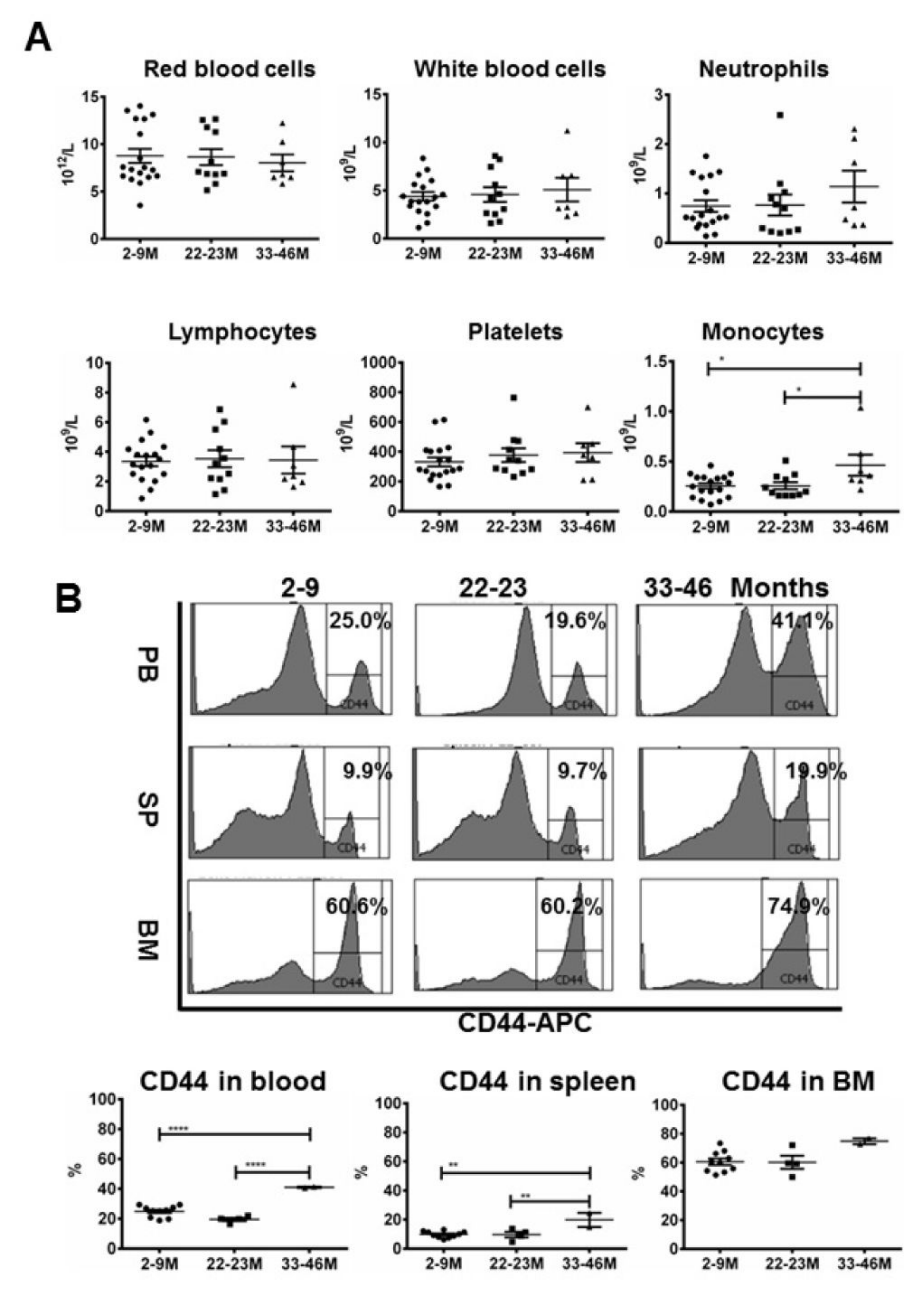
Age associated changes in hematopoietic tissues in *Peromyscus leucopus* mice. Young (2–9 months, N=18), middle (22–23 months, N=11) and old (33–46 months, N=7) PL mice were bled from orbital sinus to measure complete blood counts and were then euthanized to extract cells from spleen and BM. (A) There were no changes with age in red blood cells, white blood cells, neutrophils, lymphocytes or platelets but there was a significantly higher number of monocytes in old than in young and middle-age animals. (B) Anti-mouse CD44 antibody was able to stain positive cell populations in peripheral blood, spleen, and BM in PL mice. The proportions of CD44^+^ cells were significantly higher in old than in young and middle-age PL mice. *, P< .05; **, P< .01; ****, P< . 0001.

A significant change we observed was a higher number of circulating monocytes in old PL mice (0.46 ± 0.07 × 10^9^/L) relative to young (0.26 ± 0.06 × 10^9^/L) and middle-age (0.26 ± 0.07 × 10^9^/L) animals, suggesting a potential age-related hematopoietic skewing toward myeloid lineage (Figure 1A). There were no age effects on red blood cells, white blood cells, neutrophils, lymphocytes, or platelets (Figure 1A). Our observation was consistent with age-related myeloid lineage skewing reported in the MM mouse model, in which aging is associated with increased self-renewal and reduced lymphoid potential in hematopoietic stem cells [8]. In human studies, aging is also associated with higher blood monocyte counts with a specific expansion of the more mature non-classical monocytes [18]. In elderly individuals, CD34^+^CD38^+^CD90^−^CD45RA^+/−^CD10^−^ and CD34^+^CD33^+^ myeloid progenitor cells persisted while CD34^+^CD38^+^CD90^−^CD45RA ^+^CD10^+^ and CD34^+^CD19^+^ B-lymphoid progenitor cells decrease, indicating age-associated lineage bias toward myeloid differentiation [19,20]. Thus, increased monocytes with enhanced myeloid differentiation is a shared aging biomarker.

Another change we observed in old PL mice was an increase in the proportion of CD44^+^ cells in nucleated cells from peripheral blood (25.0 ± 2.0%, 19.6 ± 2.6% and 41.1 ± 2.9% for young, middle and old animals), spleen (9.9 ± 1.9%, 9.7 ± 2.5% and 19.9 ± 2.8%), and BM (60.7 ± 4.4%, 60.2 ± 5.7% and 74.9 ± 6.4%, Figure 1B). In general, the proportion of CD44^+^ cells was BM>peripheral blood>spleen regardless of age (Figure 1B). Age difference in CD44 expression was statistically significant in peripheral blood (P<0.0001) and spleen (P<0.01), and was marginally significant in the BM (P<0.07). Our finding of age-related CD44 up-regulation in PL mice was in agreement with observations from MM and other animal models. The utility of increased CD44 expression as an aging biomarker is originally characterized in the MM mouse models [9–12]. In a rat model of experimental autoimmune encephalomyelitis (EAE), increased proportion of splenic CD44^+^CD4^+^ T cells is observed in aged rats, leading to entrapping of activated CD4^+^ cells in the spleen to restrict EAE development [15]. In a burn-associated wound-healing rat model, CD44 expression on the wound bed is significantly increased in aged rats relative to young rats, which correlates to a delayed wound healing [21]. In aging endothelial cells, epigenetic activation of CD44 expression leads to de-methylation in the promoter regions of the CD44 gene to cause high CD44 expression on cell surface that increases adhesion to monocytes [22]. Thus, age-related CD44 up-regulation not only serves as a phenotypic marker but also exerts interference with normal cellular activities. We were unable to pinpoint age-related CD44 up-regulation to specific hematopoietic cell types in PL mice because antibodies (such as CD3, CD4, and CD8) specific for MM and human do not recognize hematopoietic cells from PL mice [6].

We also examined BM cellularity and morphology in PL mice but found no difference among the three age groups. BM cells from PL mice were capable of forming hematopoietic colonies when cultured in vitro in MethoCult media supplemented with hematopoietic cytokines for MM animals, however, the number of colony-forming cells was few, especially in young mice, and the time of colony formation was delayed in PL mice (Data not shown). A possible explanation is that hematopoietic cytokines and their receptors might be different between MM and PL mice.

Despite shared features of appearance, PL mice differ drastically from MM mice in their higher physical agility [17], longer lifespan [4], and shorter telomeres [7]. PL mice belong to Genus *Peromyscus* of Subfamily *Sigmodontinae* while MM mice belong to Genus *Mus* of Subfamily *Murinae* in the taxonomic hierarchy, which means that these two species have been separated from each other for millions of years.

In summary, we identified two aging biomarkers in PL mice: 1) elevated circulating monocytes; and 2) up-regulated CD44 expression on hematopoietic cells. Both biomarkers were detected at old age of 33–44 months, not at middle age of 22–23 months. The delayed onset of both biomarkers in PL mice, relative to those in conventional MM mice, was likely related to the long lifespans in PL mice.
